# GHRH and insulin hypersecretion from a pancreatic neuroendocrine tumor in multiple endocrine neoplasia type 1

**DOI:** 10.1210/jcemcr/luag002

**Published:** 2026-04-16

**Authors:** Elisa Lamback, Daniel Alves Bulzico, Delmar Muniz Lourenço, Alexandre Vasiljevic, Gerald Raverot, Mônica R Gadelha

**Affiliations:** Neuroendocrinology Research Center, Endocrinology Section, Medical School and Hospital Universitário Clementino Fraga Filho, Universidade Federal do Rio de Janeiro, Rio de Janeiro 21941-617, Brazil; Neuropathology and Molecular Genetics Laboratory, Instituto Estadual do Cérebro Paulo Niemeyer, Rio de Janeiro 20231-092, Brazil; Neuroendocrine Unit, Instituto Estadual do Cérebro Paulo Niemeyer, Rio de Janeiro 20231-092, Brazil; Endocrine Oncology Unit, Brazilian National Institute of Cancer, Rio de Janeiro 20230-130, Brazil; Endocrine Genetics Unit, Laboratory of Cellular and Molecular Endocrinology (LIM-25), Division of Endocrinology and Metabolism, Hospital das Clínicas, University of São Paulo School of Medicine, São Paulo 05403-010, Brazil; Division of Endocrine Oncology, Institute of Cancer of the State of São Paulo, University of São Paulo School of Medicine, São Paulo 01246-000, Brazil; Centre de Pathologie Est, Groupement Hospitalier Est, Hospices Civils de Lyon, Lyon 69003, France; Cancer Research Center of Lyon, Inserm U1052, Claude Bernard Lyon 1 University, Lyon 69373, France; Endocrinology Department, Reference Center for Rare Pituitary Diseases HYPO, “Groupement Hospitalier Est” Hospices Civils de Lyon, Bron 69500, France; Neuroendocrinology Research Center, Endocrinology Section, Medical School and Hospital Universitário Clementino Fraga Filho, Universidade Federal do Rio de Janeiro, Rio de Janeiro 21941-617, Brazil; Neuropathology and Molecular Genetics Laboratory, Instituto Estadual do Cérebro Paulo Niemeyer, Rio de Janeiro 20231-092, Brazil; Neuroendocrine Unit, Instituto Estadual do Cérebro Paulo Niemeyer, Rio de Janeiro 20231-092, Brazil

**Keywords:** multiple endocrine neoplasia type 1, growth hormone–releasing hormone, insulinoma, pancreatic neuroendocrine tumors, acromegaly

## Abstract

Acromegaly caused by ectopic growth hormone–releasing hormone (GHRH)-secreting neuroendocrine tumor (NET) is extremely rare, with cosecreting NETs even more seldom. We report a case of a female patient who presented with primary hyperparathyroidism (pHPT) and a GHRH- and insulin cosecreting pancreatic NET (pNET) and genetically confirmed multiple endocrine neoplasia type 1 (MEN1), within an undiagnosed family with various MEN1-related NETs. Due to the diagnosis of MEN1, screening for pituitary tumor was performed with biochemical evidence of acromegaly. Sellar magnetic resonance imaging revealed a sellar lesion, which was excised and compatible with somatotroph hyperplasia. Postoperatively, the patient developed severe hypoglycemia requiring hospitalization and the pNET was removed. Histopathology confirmed GHRH and insulin secreting pNET. Ectopic acromegaly in MEN1 is exceedingly rare. Patients with MEN1 often present with multiple pNETs, which may exhibit multihormonal secretion and frequently cosecrete GHRH and insulin in MEN1, while hypoglycemia may not be manifested possibly due to GH and insulin's counteractive effects on glucose metabolism.

## Introduction

Acromegaly is commonly caused by growth hormone (GH)-secreting pituitary tumors, but can also result from ectopic secretion of GH-releasing hormone (GHRH) by neuroendocrine tumor (NET) (accounting for <1% of cases) [1] and even rarer by ectopic GH secretion [2]. It can be present in multiple endocrine neoplasia type 1 (MEN1), a rare autosomal dominant syndrome caused by pathogenic germline variants in the *MEN1* gene [3, 4]. Primary hyperparathyroidism (pHPT) is the most common manifestation, present in up to 100% of patients, followed by duodenopancreatic NET (30%-90%), and pituitary tumors (30%-40%) [3-5]. In MEN1, GHRH-secreting tumors are exceedingly rare, with cosecreting NETs even more seldom [6].

## Case presentation

A 41-year-old White female, Jehovah's witness, presented with pHPT, which had been treated surgically at ages 32 and 35. Histopathological examination revealed asynchronous multiglandular parathyroid disease adenomas. At age 36, during tomographic assessment of a thoracic lipoma, incidental pancreatic lesions were identified.

## Diagnostic assessment

**Figure 1:**
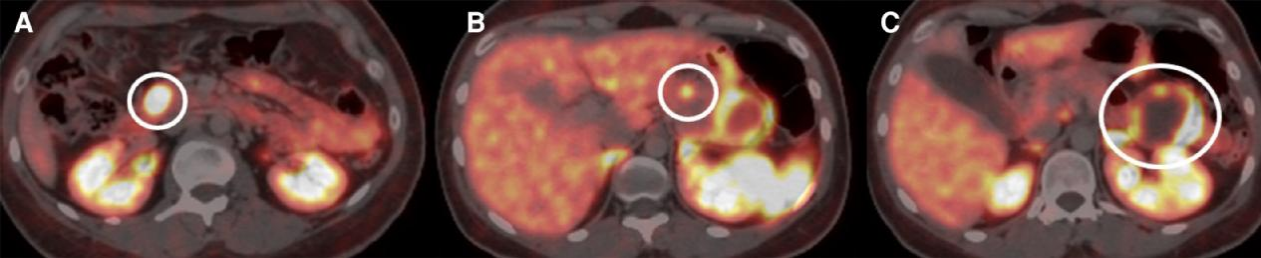
Axial views of ^68^Ga-DOTATOC PET/CT exhibiting neuroendocrine tumors on the head of the pancreas (A, white circle), body (B, white circle), and tail (C, white circle).

Biopsy of these lesions was consistent with a pancreatic NET (pNET). Functional imaging with Gallium-68 DOTA-D-Phe1-Try3-Octreotide positron emission tomography/computed tomography (^68^Ga-DOTATOC PET/CT) demonstrated increased somatostatin receptor expression in 3 pancreatic tumors: a 47- × 47-mm nodule in the tail (maximum standardized uptake value, SUVmax 26.2), a 16- × 13-mm in the head (SUVmax 37.9), and an irregular mass in the body (SUVmax 10) (Fig. 1). The patient was asymptomatic, with no clinical evidence of hormone hypersecretion or mass effect. Chromogranin A was within normal limits at 17 µg/L (international system of unit, SI: 38.5 nmol/L) (reference value <100 µg/L; SI: <226 nmol/L).

She was clinically diagnosed with MEN1 due to the presence of recurrent pHPT for asynchronous multiglandular parathyroid disease and multifocal pNETs. Germline genetic testing identified a pathogenic variant in *MEN1* (c.1348T > G, p.L413R). Family screening of the patient's mother (aged 73), brother (aged 50), sister (aged 51), and daughter (aged 3) was negative. The patient's father, likely a carrier, died at age 67 of a metastatic pancreatic tumor. A first-degree cousin on her father's side has a lactotroph tumor and pHPT, and her son has a nonfunctioning pituitary tumor. Both have the same pathogenic variant as the patient has.

**Figure 2:**
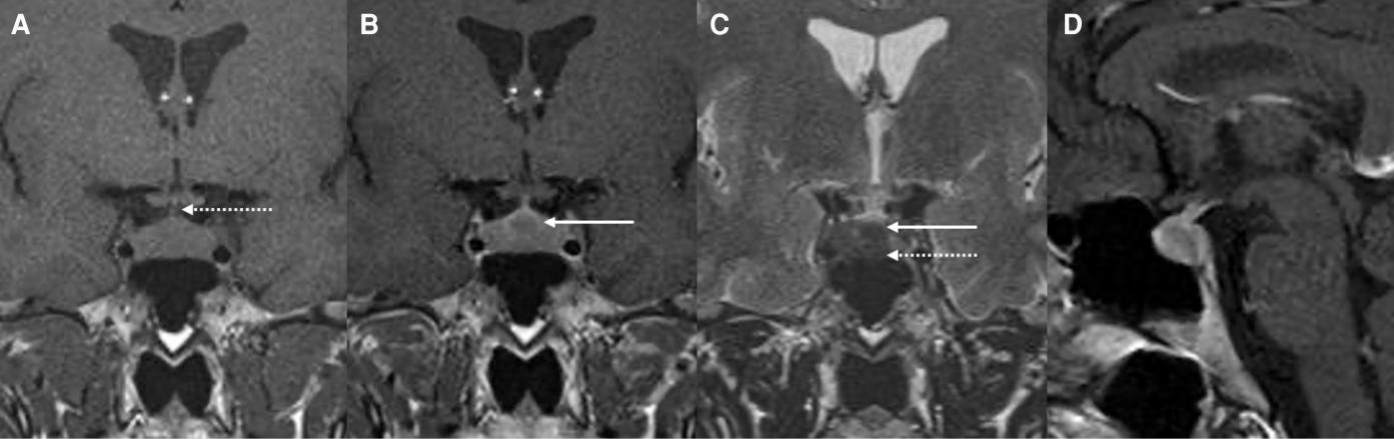
Sellar magnetic resonance imaging on A, coronal T1; B, coronal T1 post contrast; C, coronal T2; and D, sagittal T1 post contrast showing a symmetrical pituitary gland enlargement with convex superior margin (dotted arrow in A), and marked T2 hypointensity (dotted arrow in C), which was initially considered to be a pituitary adenoma because of a misinterpreted central nodule (white arrow in B and C).

Due to the diagnosis of MEN1, screening for pituitary tumor was performed at age 38. Mild hyperprolactinemia (26 µg/L; reference range, 5-23 µg/L), along with biochemical evidence of acromegaly (elevated insulin-growth factor 1 [IGF-I] 701 ng/mL [SI: 91.6 nmol/L] [reference range, 63-223 ng/mL (SI: 8.2-29.2 mL)]), and random GH concentration of 7.4 ng/mL. No physical signs of acromegaly were observed on examination. Sellar magnetic resonance imaging (MRI) revealed a central sellar nodule of 9 × 8 × 6 mm, initially considered to be a pituitary adenoma (Fig. 2).

## Treatment

**Figure 3:**
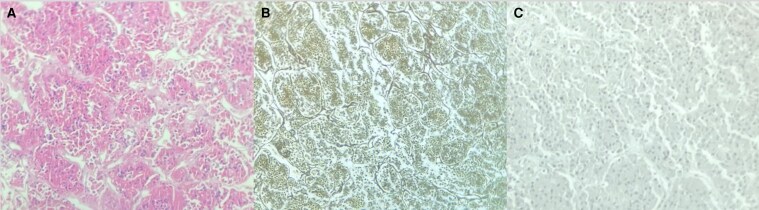
Histology slides of the pituitary specimen showing normal adenohypophyseal tissue exhibiting predominance of epithelial cells with eosinophilic cytoplasm (A, magnification 100×), enlarged acini with intact interacinar framework highlighted by the reticulin fiber staining (B, magnification 100×), with weak cytoplasmatic positivity for growth hormone (C, magnification 100×), characterizing somatotroph hyperplasia.

The patient underwent transsphenoidal surgery for excision of the pituitary lesion. Ten days postoperatively, she developed severe hypoglycemia (capillary glucose 40-50 mg/dL, SI: 2.2-2.8 mmol/L) requiring hospitalization. Histopathological examination of the pituitary tissue revealed somatotroph hyperplasia (Fig. 3). Sellar MRI was reviewed and showed a symmetrical pituitary gland enlargement with convex superior margin, and T2 hypointensity, suggestive of pituitary hyperplasia (see Fig. 2). Postoperative GH and IGF-I concentrations decreased, but did not normalize (GH 3.7 ng/mL; IGF-I 419 ng/mL [SI: 54.8 nmol/L]). Plasma GHRH measurement was not available in the patient's country (Brazil).

Due to severe hypoglycemia, intravenous glucose infusion was required, and the possibility of partial pancreatectomy was discussed. Abdominal CT revealed the pNETs, as well as a 2.0-cm hypodense image in the endometrial cavity. Serum β-human chorionic gonadotropin concentration was elevated (25 423.2 mIU/mL [reference <5 mIU/mL]) and transvaginal ultrasonography confirmed a previously unknown intrauterine pregnancy. Following a complex and difficult discussion with the patient and her family, surgical resection of the pNETs was pursued due to life-threatening hypoglycemia, even though pregnancy was estimated at gestational age 6 weeks.

**Figure 4:**
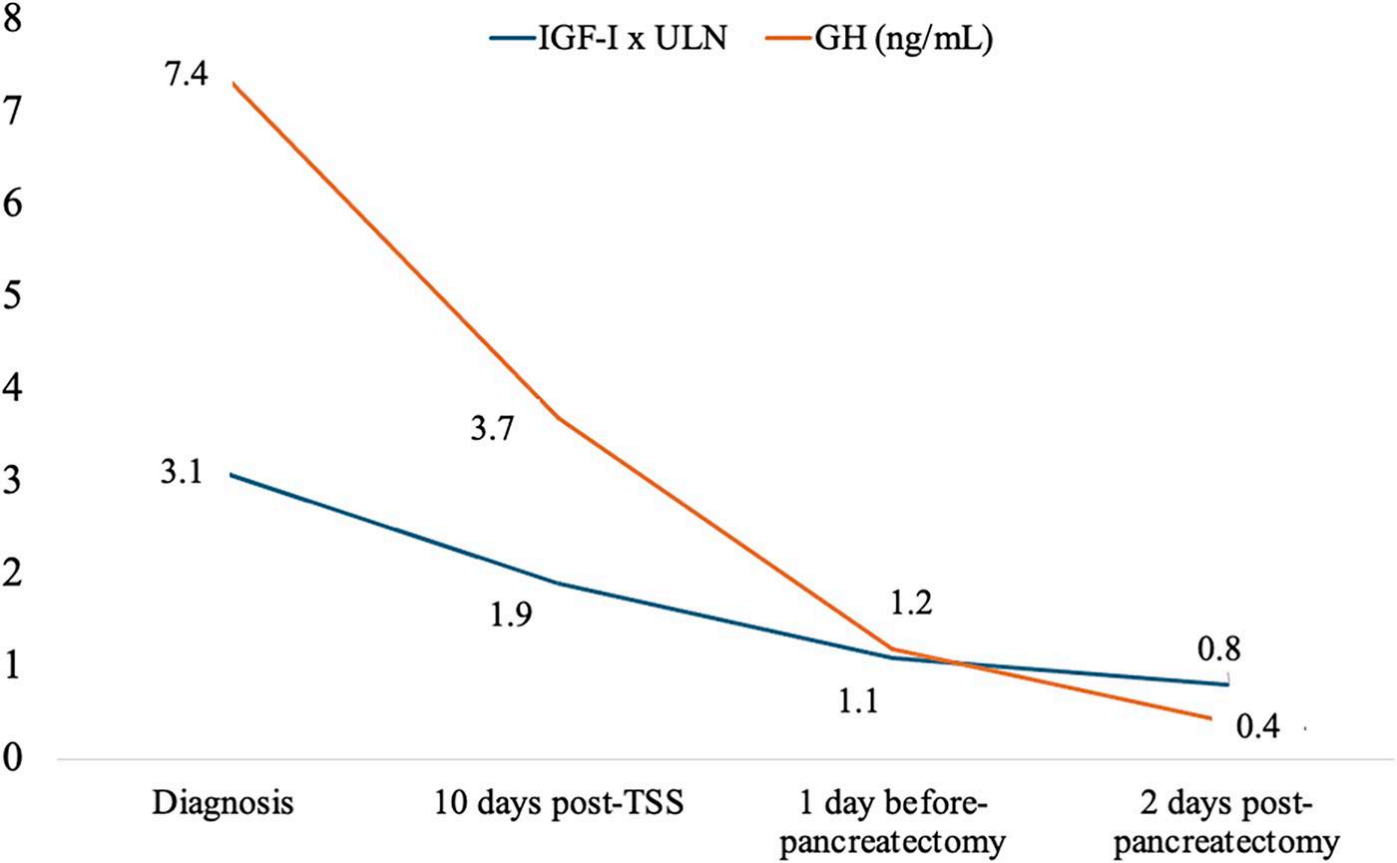
Growth hormone (GH) and insulin-like growth factor-I (IGF-I) levels at diagnosis, after transsphenoidal surgery (TSS), pre pancreatectomy and post pancreatectomy. IGF-I is expressed in × ULN (times the upper limit of normal range).

One day before surgery, GH and IGF-I concentrations were 1.23 ng/mL and 254 ng/mL (SI: 33.2 nmol/L), respectively. Two days postoperatively, GH decreased to 0.38 ng/mL and IGF-I to 180 ng/mL (SI: 23.5 nmol/L), with normalization of blood glucose levels (Fig. 4).

**Figure 5:**
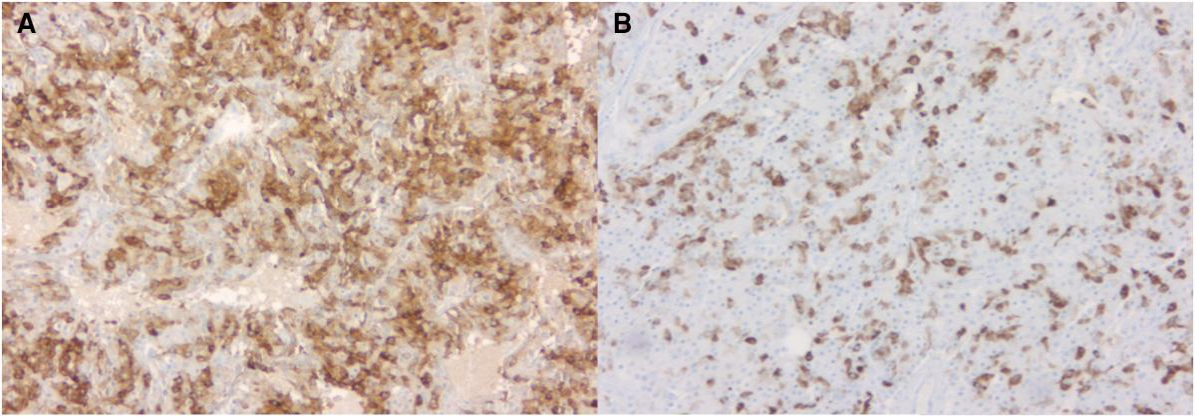
Immunohistochemistry of the cosecreting growth hormone–releasing hormone (GHRH) and insulin-pancreatic neuroendocrine tumor exhibiting GHRH (A, magnification 100×) and insulin (B, magnification 100×) positive pancreatic neuroendocrine tumor.

Histopathological analysis showed grade 1 pNETs with one tumor expressing both GHRH and insulin (Fig. 5). α Thalassemia/mental retardation syndrome X-linked (ATRX) immunostaining was preserved (positive nuclear staining) in this pNET. In the remaining 2 pNETs, technical artifacts compromised the interpretation of the immunohistochemistry.

## Outcome and follow-up

At 26 weeks of gestation, the patient was diagnosed with gestational diabetes, which was managed with dietary modifications. Cesarean delivery was performed at term due to the presence of multiple and large uterine myomas. She gave birth to a healthy girl weighing less than 4 kg, who is developing normally.

Due to rising serum calcium and PTH concentrations, a subtotal parathyroidectomy was performed, at age 40 years, with excision of all parathyroid glands except for half of one macroscopically normal gland, along with prophylactic thymectomy. At last follow-up, ^68^Ga-DOTATOC PET/CT identified 5 small lesions in the remnant pancreatic parenchyma. The largest one was a 1.2-cm lesion located in the pancreatic head (SUVmax = 18.2). Additionally, a 1.7-cm pulmonary nodule (SUVmax = 4.6) was identified. Biochemical markers including chromogranin A, vasoactive intestinal peptide, glucagon, gastrin, glucose, insulin, and IGF-I remain within normal limits.

Retrospective review of the patient’s medical records revealed biochemical evidence of hyperinsulinemia as early as age 31, with a random glucose level of 67 mg/dL (SI: 3.7 mmol/L) and insulin of 40 mIU/mL (SI: 277.8 pmol/L)], and, at age 36, glucose concentration lower than 55 mg/dL (SI: 3.0 mmol/L) (value 51 mg/dL [SI: 2.8 mmol/L]), with inappropriately high insulin at 12 mIU/mL (SI: 83.3 pmol/L) (reference > 3 mIU/mL [20.8 pmol/L]) and C-peptide at 3.1 ng/mL (SI: 1.0 nmol/L) (reference > 0.6 ng/mL [SI: 0.2 nmol/L]), confirming hyperinsulinemic hypoglycemia, although she was asymptomatic at that time.

## Discussion

In MEN1, GHRH-secreting NETs have been described in 26 pancreatic cases (Table 1**)**, including the present case, and in 1 thymic NET, whereas none have been reported with pulmonary origin [7-35]. The majority were male (16 men, 10 women), with median age at presentation of MEN1 of 33 years (range, 7-67 years).

**Table 1:** Patients with acromegaly from pancreatic neuroendocrine tumor secreting growth hormone–releasing hormone in the context of multiple endocrine neoplasia type 1

Publication	Sex	Age of 1st MEN1 presentation, y	pHPT	Pituitary tumor	Plasma or serum hormonal secretion	Family history of NET	Known MEN1	*MEN1* variant
Aida, 1977 (35) Sasaki, 1985 (11) Sasaki, 1989 (10)	F	39	Yes: hyperplasia	Yes: tumor	GH Calcitonin	Yes	No	NA*^a^*
Guillemin, 1982 (12) Sassolas, 1983 (13) Berger, 1984 (14)	M	55	Yes	No: pituitary hyperplasia	GH Glucagon Somatostatin PP	NA	NA	NA*^a^*
Wilson, 1984 (15) Wilson, 1986 (16)	F	19	Yes	Yes	GHRH Gastrin	NA	NA	NA*^a^*
Ch'ng, 1985 (17) Ch'ng, 1986 (18)	F	25	Yes	No	GHRH Gastrin	Yes	NA	NA*^a^*
Ramsay, 1988 (19)	F	28	Yes	No	GH, GHRH Insulin	No	No	NA*^a^*
Sano, 1987 (20) Yamasaki, 1988 (21) Shintani, 1995 (22)	M	31	Yes: hyperplasia	Yes: somatotroph tumor and hormone-negative tumor	GH, GHRH Insulin	Yes	Yes	NA*^a^*
Price, 1992 (24)	F	42	Yes	No	GH, GHRH Calcitonin Insulin	Yes	Yes	NA*^a^*
Bertherat, 1994 (25)	M	23	Yes: hyperplasia	Yes: gonadotroph tumor and somatotroph hyperplasia	GH, GHRH Insulin	Yes	Yes	NA*^a^*
Liu, 1996 (23)	M	51	Yes: adenoma	No: somatotroph hyperplasia	GH, GHRH	Yes	No	NA*^a^*
Suga, 2002 (26)	M	27	Yes	Yes: tumor	GH PP	No	No	NA*^a^*
Biermasz, 2007 (27)	M	50	Yes: adenoma	No: somatotroph hyperplasia	GH, GHRH PP	No	No	Exon 2
Sugihara, 2007 (28)	M	31	Yes: hyperplasia	No	GH, GHRH	Yes	No	NA
Weiss, 2011 (29)	F	46	Yes: hyperplasia	Yes: lactotroph tumor and somatotroph hyperplasia	GH, GHRH	Yes	No	c.152_160del9
Garby, 2012 (30)	F	35	Yes	Yes	GH, GHRH Insulin	NA	NA	1325delG, exon 9
Garby, 2012 (30)	M	37	Yes	No	GHRH Gastrin	Yes	Yes	1325delG, exon 9
Garby, 2012 (30)	M	14	No	No: somatotroph hyperplasia	GH, GHRH	NA	NA	NA
Garby, 2012 (30)	F	34	Yes	Yes: lactotroph tumor and somatotroph hyperplasia	GH, GHRH	NA	NA	1650insC, exon 10
Garby, 2012 (30)	M	47	No	Yes: lactotroph tumor and somatotroph hyperplasia	GH, GHRH PP	NA	NA	NA
Garby, 2012 (30)	M	67	Yes	No	GH, GHRH Gastrin	NA	NA	1607delA, exon 10
Garby, 2012 (30)	M	17	No	Yes	GH, GHRH Insulin	NA	NA	NA
Garby, 2012 (30)	F	34	No	No	GH, GHRH	NA	NA	duplication exons 5-10
Saleem, 2012 (31)	M	36	No	Yes	GH, GHRH	NA	NA	NA
Sala, 2013 (32)	M	18	Yes: hyperplasia	No	GH, GHRH	Yes	No	c.207delC
Koivikko, 2022 (33)	M	22	Yes	No	GH, GHRH	Yes	Yes	c.466G > C
Srirangam Nadhamuni, 2021 (34)	M	7	Yes: hyperplasia	Possible 3 mm	GH, GHRH Insulin	Yes	Yes	c.249_252delGTCT
Present case	F	32	Yes: adenomas	No: somatotroph hyperplasia	GH Insulin	Yes	No	c.1348T > G

Abbreviations: F, female; GH, growth hormone; GHRH, growth hormone–releasing hormone; M, male; *MEN1*, multiple endocrine neoplasia type 1; NA, not available; NET, neuroendocrine tumor; pHPT, primary hyperparathyroidism; PP, pancreatic polypeptide.

^*a*^Cases reported before discovery of the *MEN1* gene in 1997.

Patients with MEN1 can have multiple duodenopancreatic NETs seen macroscopically, ranging from 0 to 13 tumors, depending on the tumor's functionality and the patient's age at diagnosis [36]. However, microscopic analysis often reveals many more due to the disease's multifocal nature, as demonstrated in several reports [28, 29, 32, 33], even in young adults and adolescents [37, 38]. It remains unclear which tumors progress, though this may involve secondary mutations, such as those affecting the death domain-associated protein (*DAXX*) and *ATRX* genes [3], which was absent in one of our patient's pNETs.

These pNETs may exhibit multihormonal secretion either across different tumors within the same pancreas or even within a single tumor. The hormonal staining of pNETs in MEN1 vary greatly. Although not all hormones were assessed in every case, GHRH-pNETs cosecrete other hormones, with insulin being the most common cosecretion (9 cases), followed by GHRH-secreting NETs cosecreting somatostatin (8 cases), glucagon (7 cases), calcitonin (5 cases), pancreatic polypeptide (5 cases), gastrin (4 cases), or vasoactive intestinal polypeptide (1 case) (Table 2). pNETs can cosecrete GHRH and insulin within the same tumor, or secrete GHRH and insulin in distinct NETs within the same patient in a synchronous or metachronous pattern (see Table 2). Although ectopic acromegaly from pNETs frequently cosecrete insulin in MEN1, in most cases, hypoglycemia is not present, possibly due to GH and insulin's counteractive effect on glucose metabolism. Hypoglycemia was manifested only during prolonged fasting [24], years after pituitary irradiation [25], and days after pituitary surgery as seen in our case. This does not seem to be related to the percentage of GHRH and insulin immunostaining (see Table 2). Of note, retrospective review of our case revealed evidence of hyperinsulinemic hypoglycemia years before partial pancreatectomy. This finding suggests that GHRH-mediated pituitary hyperplasia, through the excess of GH and IGF-I, may have contributed to counterregulating insulin-induced hypoglycemia, thereby protecting the patient from more severe episodes and overt neuroglycopenic symptoms. In addition, as commonly observed in insulinoma, adaptive mechanisms to chronic mild-to-moderate hypoglycemia likely blunted adrenergic counterregulatory manifestations.

**Table 2:** Characteristics of the growth hormone–releasing hormone neuroendocrine tumor secreting by imaging and immunocytochemistry or immunohistochemistry

Publication	No., location, and size	GHRH	Insulin	Somatostatin	Glucagon	Calcitonin	Pancreatic polypeptide	Gastrin	VIP
Aida, 1977 (35) Sasaki, 1985 (11) Sasaki, 1989 (10)	One pNET	+	NA	—	—	+++	—	+	+
Guillemin, 1982 (12) Sassolas, 1983 (13) Berger, 1984 (14)	One pNET	++	NA	+	+	NA	+	NA	NA
Wilson, 1984 (15) Wilson, 1986 (16)	One, tail	++	NA	NA	NA	NA	NA	++	NA
Ch'ng, 1985 (17) Ch'ng, 1986 (18)	One unoperated pNET	NA	NA	NA	NA	NA	NA	NA	NA
Ramsay, 1988 (19)	One in the tail and body, larger	+++	++	+	—	NA	—	—	—
	Another one in the tail and body, smaller	+++	—	—	+	NA	+	+	—
Wilson, 1984 (15) Wilson, 1986 (16)	One in the tail, larger	+	NA	+	NA	+	NA	NA	NA
	Another one in the tail, smaller	—	NA	NA	+	NA	+	NA	NA
Price, 1992 (24) Bertherat, 1994 (25) Liu, 1996 (23) Price, 1992 (24)	1st pNET	NA	+	NA	NA	NA	NA	NA	NA
	2nd pNET	NA	+	NA	NA	NA	NA	NA	NA
	3rd pNET	NA	+	NA	+	+	NA	NA	NA
	4th pNET	NA	NA	NA	+	NA	NA	NA	NA
	5th pNET	NA	NA	+	NA	NA	NA	NA	NA
Bertherat, 1994 (25) Liu, 1996 (23) Price, 1992 (24)	One in the tail	+++	+	NA	NA	NA	NA	NA	NA
	Another in the tail	+	+++	NA	NA	NA	NA	NA	NA
	One in the head	+++	+	NA	NA	NA	NA	NA	NA
	Another in the head	+	+	NA	NA	NA	NA	NA	NA
Bertherat, 1994 (25) Liu, 1996 (23) Price, 1992 (24)	One in the body, 5.0 cm	+	NA	NA	NA	NA	NA	NA	NA
	Another in the body, <1cm	+	NA	NA	NA	NA	NA	NA	NA
	A 3rd in the body, <1cm	+	NA	NA	NA	NA	NA	NA	NA
	A 4th in the body, <1cm	+	NA	NA	NA	NA	NA	NA	NA
Bertherat, 1994 (25)	One in the tail, 7.0 cm	+	NA	NA	NA	NA	NA	NA	NA
Biermasz, 2007 (27) Sugihara, 2007 (28) Weiss, 2011 (29) Garby, 2012 (30)	One in the body, 5.0 cm	NA	+	+	+	NA	NA	NA	NA
	Another in the body, <1cm	NA	+	+	+	NA	NA	NA	NA
	A 3rd in the body, <1cm	NA	+	+	+	NA	NA	NA	NA
	A 4th in the body, <1cm	NA	+	+	+	NA	NA	NA	NA
Garby, 2012 (30) Garby, 2012 (30)	One in the tail 2.2 cm	++	+	+	+	NA	+	NA	NA
	Several, uncountable, in the tail, <1cm	NA	NA	NA	+	NA	NA	NA	NA
Garby, 2012 (30) Garby, 2012 (30)	One in the tail and body, 6.0 cm	+	NA	NA	NA	NA	NA	NA	NA
	Several, uncountable, in the tail and body, <1cm	NA	NA	NA	NA	NA	NA	NA	NA
Garby, 2012 (30)	One pNET, 8.0 cm	+++	NA	NA	NA	NA	NA	NA	NA
Garby, 2012 (30)	One pNET, 5.0 cm	NA	NA	NA	NA	NA	NA	NA	NA
Garby, 2012 (30)	One pNET, 8.0 cm	+++	NA	NA	NA	NA	NA	NA	NA
Saleem, 2012 (31)	One pNET, 4.5 cm	NA	NA	NA	NA	NA	NA	NA	NA
Sala, 2013 (32)	One pNET, 1.3 cm	NA	NA	NA	NA	NA	NA	NA	NA
Koivikko, 2022 (33)	One pNET, 4.3 cm	NA	NA	NA	NA	NA	NA	NA	NA
Biermasz, 2007 (27)	One pNET, 7.0 cm	NA	NA	NA	NA	NA	NA	NA	NA
Sugihara, 2007 (28)	One pNET, 1.0 cm	NA	NA	NA	NA	NA	NA	NA	NA
Weiss, 2011 (29)	One in the head	NA	NA	NA	NA	NA	NA	NA	NA
Garby, 2012 (30) Garby, 2012 (30) Garby, 2012 (30) Garby, 2012 (30)	3 in the tail and body, 0.9-1.5 cm	+++	+	—	—	NA	NA	—	NA
		NA	NA	NA	NA	NA	NA	NA	NA
		NA	NA	NA	NA	NA	NA	NA	NA
	18 others in tail and body, <1cm	NA	NA	NA	NA	NA	+	NA	NA
Garby, 2012 (30)	One in the tail, 5.0 cm	+++	+	+	NA	+	NA	+	NA
	9 in.the body and neck, <1cm	NA	NA	NA	NA	NA	NA	NA	NA
Srirangam Nadhamuni, 2021 (34)	One in the neck, 1.0 cm	NA	+++	+	+	+	NA	NA	NA
	Another one in the head, 2.7cm	+	—	—	—	+	NA	NA	NA
	A 3rd one in the head, 0.8cm	—	—	—	+	—	NA	NA	NA
	A 4th in the head, 0.4cm	—	—	—	—	—	NA	NA	NA
	A 5th in the head, 0.3cm	—	—	—	—	—	NA	NA	NA
Present case	1 in the body, 3.5 cm	+++	+	NA	NA	NA	NA	NA	NA
	One in the tail, 4.7cm	NA	NA	NA	NA	NA	NA	NA	NA
	One in the head, 2.0cm	NA	NA	NA	NA	NA	NA	NA	NA

^+++^diffuse; ++ intense in few nests/moderate; + scattered.

Abbreviations: GHRH, growth hormone–releasing hormone; GHRH-NET, GHRH-secreting neuroendocrine tumor; micro, microadenomas; NA, not available/performed; VIP, vasoactive intestinal polypeptide.

Interestingly, insulinomas may become overt during pregnancy as increased insulin concentrations, sensitivity, and β-cell hyperplasia are observed during this period [39]. To date, only 24 cases of apparently sporadic insulinomas diagnosed during pregnancy have been reported in the literature [39]. More recently, a pregnant woman with MEN1 was reported with an atypical presentation of insulinoma, characterized by postprandial hypoglycemia during the second trimester [40]. Her symptoms remitted for several months during the puerperium, but overt hypoglycemia recurred 8 months post partum [40]. Additionally, given that almost 80% of GHRH-secreting pNETs are associated with MEN1, a diagnosis of MEN1 should always be considered in GHRH-secreting pNET [1, 30]. Moreover, a family history of NET was documented in 13 of 16 (81%) cases, with known MEN1 diagnosis in 6 families (see Table 1).

Regarding pituitary involvement, 7 of 26 (27%) patients with MEN1 and GHRH-secreting pNET also had a pituitary tumor confirmed on histopathology (see Table 1). A patient can have pituitary hyperplasia from the ectopic GHRH source and an adenomatous transformation [7]. Since pituitary incidentalomas are relatively common in the general population (with an estimated prevalence of 10%), and because increased GH and prolactin can occur in the context of GHRH-secreting NETs, establishing an accurate differential diagnosis is crucial [41, 42]. Prolactin secretion can be stimulated by GHRH, as demonstrated by its mild, dose-dependent increase following GHRH injections [41]. This mechanism may even lead to a misdiagnosis of microprolactinoma. Therefore, because ectopic and pituitary acromegaly are clinically indistinguishable and cannot be differentiated by GH and prolactin measurements alone, plasma GHRH assessment is recommended [30, 42]. Alternatively, MRI characteristics, such a symmetrical and enlarged pituitary gland with a convex superior margin, homogeneous contrast enhancement, and T2 hypointensity, can favor somatotroph hyperplasia due to ectopic GHRH NET over pituitary tumor [43].

The pathogenic variant p.L413R identified in the present case has previously been reported in 2 other Brazilian MEN1 families, in whom mitochondrial DNA and Y-chromosome haplotype analyses excluded a founder effect [44, 45]. Genealogical review revealed no apparent relationship between these families and the present patient. Notably, no MEN1 cases carrying this variant have been reported outside Brazil. Importantly, segregation of the variant in the previously described families, its absence from population genomic databases—including the local ABraOM—and in silico prediction analyses support its pathogenicity.

## Learning points

- Ectopic acromegaly in MEN1 is exceedingly rare—to date, only 26 cases of pNETs and 1 thymic NET have been identified.
- Patients with MEN1 often present with multiple pNETs, which may exhibit multihormonal secretion either across different tumors within the same pancreas or even within a single tumor.
- Ectopic acromegaly from pNETs frequently cosecrete insulin in MEN1, while hypoglycemia may not be manifested possibly due to GH and insulin's counteractive effects on glucose metabolism.

## Contributors

E.L. reviewed the literature and drafted the manuscript. D.M.L. was responsible for the next generation sequencing of the index case. A.V. was involved in immunohistochemistry of GHRH and insulin. D.A.B., D.M.L., G.R., and M.R.G. critically reviewed the final draft. All authors reviewed and approved the final draft.

## Data Availability

Original data generated and analyzed during this study are included in this published article.

## Abbreviations

GH: growth hormone
GHRH: growth hormone–releasing hormone
MEN1: multiple endocrine neoplasia type 1
MRI: magnetic resonance imaging
NET: neuroendocrine tumor
PET/CT: positron emission tomography/computed tomography
pHPT: primary hyperparathyroidism
pNET: pancreatic neuroendocrine tumor
SUVmax: maximum standardized uptake value
